# Application of ECIS to Assess FCCP-Induced Changes of MSC Micromotion and Wound Healing Migration

**DOI:** 10.3390/s19143210

**Published:** 2019-07-21

**Authors:** Sheng-Po Chiu, Yu-Wei Lee, Ling-Yi Wu, Tse-Hua Tung, Sofia Gomez, Chun-Min Lo, Jia-Yi Wang

**Affiliations:** 1 Graduate Institute of Medical Sciences, College of Medicine, Taipei Medical University, Taipei 11031, Taiwan; 2 Division of Endocrinology and Metabolism, Department of Internal Medicine, Tri-Service General Hospital Songshan Branch, National Defense Medical Center, Taipei 11490, Taiwan; 3 Department of Biomedical Engineering, National Yang-Ming University, Taipei 11221, Taiwan; 4 Department of Physiology, School of Medicine, College of Medicine, Taipei Medical University, Taipei 11031, Taiwan

**Keywords:** ECIS, human mesenchymal stem cells (hMSCs), mitochondria, carbonyl cyanide 4-(trifluoromethoxy)phenylhydrazone (FCCP), micromotion, wound healing migration

## Abstract

Electric cell-substrate impedance sensing (ECIS) is an emerging technique for sensitively monitoring morphological changes of adherent cells in tissue culture. In this study, human mesenchymal stem cells (hMSCs) were exposed to different concentrations of carbonyl cyanide 4-(trifluoromethoxy)phenylhydrazone (FCCP) for 20 h and their subsequent concentration-dependent responses in micromotion and wound healing migration were measured by ECIS. FCCP disrupts ATP synthesis and results in a decrease in cell migration rates. To detect the change of cell micromotion in response to FCCP challenge, time-series resistances of cell-covered electrodes were monitored and the values of variance were calculated to verify the difference. While Seahorse XF-24 extracellular flux analyzer can detect the effect of FCCP at 3 μM concentration, the variance calculation of the time-series resistances measured at 4 kHz can detect the effect of FCCP at concentrations as low as 1 μM. For wound healing migration, the recovery resistance curves were fitted by sigmoid curve and the hill slope showed a concentration-dependent decline from 0.3 μM to 3 μM, indicating a decrease in cell migration rate. Moreover, dose dependent incline of the inflection points from 0.3 μM to 3 μM FCCP implied the increase of the half time for wound recovery migration. Together, our results demonstrate that partial uncoupling of mitochondrial oxidative phosphorylation reduces micromotion and wound healing migration of hMSCs. The ECIS method used in this study offers a simple and sensitive approach to investigate stem cell migration and its regulation by mitochondrial dynamics.

## 1. Introduction

Human mesenchymal stem cells (hMSCs) from different sources have gained great interest in many area of tissue engineering and regenerative medicine, as they are multipotent stem cells with the capacity to differentiate into mesodermal (osteocytes, adipocytes, and chondrocytes), ectodermal (neurocytes), and endodermal lineages (hepatocytes) [1]. In addition to proliferation and differentiation, the migratory ability of stem cells is required for embryogenesis, homoeostasis, repair of adult tissues, and regeneration [2,3]. Many studies have highlighted that the systemic infusion of stem cells for therapeutic applications requires efficient migration and homing to the target site [4]. Cell migration involves regulation of metabolic energy supplies for reorganization of actin microfilaments and myosin motor function. Adenosine triphosphate (ATP) is the energy currency of life and it provides energy to drive many biological processes in living cells. Mitochondria are the powerhouse of the cell to produce the majority of ATP through oxidative phosphorylation (OXPHOS), which refers to the redox reactions involving the electron transport chain and the ATP synthase complexes. These activities require a complex series of events to occur in a regulated and integrated fashion. For example, succinate is a key intermediate in the tricarboxylic acid cycle and has been demonstrated to induce mitochondrial ROS (reactive oxygen species) and promotes hMSC migration [5]. Moreover, hypoxia or hypoxia-inducible factor 1 alpha (HIF-1α) stabilization improves several MSC functions, including cell adhesion, proliferation, and migration [6,7]. Mitochondrial metabolism in regulating hMSC proliferation and differentiation has been extensively studied using the uncoupler, carbonyl cyanide m-chlorophenylhydrazone (CCCP) or carbonyl cyanide 4-(trifluoromethoxy) phenylhydrazone (FCCP) [8,9]. Both CCCP and FCCP are uncouplers or uncoupling agents which can move protons across lipid bilayers, decrease the proton gradient in the inner mitochondrial membrane, uncouple the electron transport chain in the OXPHOS pathway, and reduce ATP production. It has been reported that oxygen consumption increase significantly during adipogenic differentiation of hMSCs, and that reducing mitochondrial respiration by inhibition of the mitochondrial electron transport chain significantly suppresses adipogenic differentiation [9]. However, the dose-dependent effect of these uncouplers on hMSC migration via regulating mitochondrial dysfunction remains unclear.

Recent studies have reported that the interplay between OXPHOS and glycolysis have relevance to stem cell proliferation and differentiation [10,11]. Glycolysis is an oxygen-independent metabolic pathway which converts glucose to pyruvate via a series of intermediate metabolites. For example, human pluripotent stem cells (hPSCs) contain underdeveloped and metabolically inactive mitochondria and produce more lactate than differentiated cells, indicating that glycolysis supplants OXPHOS and play a major role in ATP production in hPSCs [12]. In addition, the differentiation of pluripotent stem cells (PSCs) usually couples with a transition from a mainly glycolytic metabolism towards an OXPHOS based metabolism [13,14]. Exposure of mouse embryonic stem cells (mESCs) to antimycin A, an inhibitor to cause the disruption of the entire electron transport chain in the OXPHOS, enhances cell pluripotency and inhibits their neuronal differentiation even when subjected to a specific differentiation protocol [15]. However, inducing mitochondrial uncoupling in human embryonic stem cells (hESCs) using different concentrations of CCCP, ranging from 1 μM to 12 μM, results in a decrease in intracellular ATP levels and reduces proliferation rates [16]. This study demonstrates that normal mitochondrial function is essential in regulating proliferation of undifferentiated ESCs and early differentiation [16]. The discrepancies between the above-mentioned studies might be due to different degrees of OXPHOS inhibition caused by different concentrations of CCCP. Thus, there is an emerging interest in understanding the functional role of mitochondria in stem cell biology, given the potential applications of stem cells in regenerative medicine [17,18]. However, previous studies analyzing energy metabolism of stem cells have focused primarily on proliferation and differentiation. Few studies have assessed the role of energy metabolism in controlling other important stem cell phenotypes such as cellular migration [5].

Cell migration in tissue culture has been extensively observed, as many adherent cells exhibit the ability to crawl on culture dish or coverglass and perform other subtle motions. In addition to locomotion, cell migration may take the form of membrane ruffling and the extension of cytoplasm in the form of blebs and lamellipodia [19]. When cells are exposed to a chemical compound such as FCCP, the effect may act at any point in the series of migratory events and alter the migration measurement. In this aspect, measurements of complex in vitro cell migration need more quantitative method to analyze the alteration of cell morphology and motility. Electric cell-substrate impedance sensing (ECIS) is a highly versatile impedance-based method, providing abundant information regarding changes in cell-substrate interactions and of cell morphology [20,21,22,23]. Fluctuations in impedance measured with this device have experimentally been related to cell micromotion [24,25,26,27]. Recently, we have applied ECIS wound-healing assay to investigate the cytotoxic effects of andrographolide on U-87 MG glioblastoma cell migration [28]. In this study, we used ECIS to sensitively detect the effect of different low levels of FCCP on hMSC micromotion and wound healing migration. In parallel with the ECIS measurements, the oxygen consumption rate (OCR) and extracellular acidification rate (ECAR) were measured using Seahorse XF-24 extracellular flux analyzer to verify the results. We also tested the feasibility of utilizing the ECIS method to obtain meaningful migration data that is correlated with the OXPHOS inhibition caused by FCCP and can be used in assessing energy metabolism of stem cells.

## 2. Materials and Methods

### 2.1. Cell Culture

Human mesenchymal stem cells (hMSCs) isolated from Wharton’s jelly of the umbilical cord were purchased from ATCC (PCS-500-010). Cells were cultured in Dulbecco’s modified Eagle’s medium (Invitrogen, CA, USA) supplemented with 2% fetal bovine serum (Thermo, Logan, UT, USA) under 5% CO_2_ at 37 °C. Upon reaching 70–80% confluence, adherent hMSCs were detached from culture flasks by adding HyQtase (Thermo-Fischer Scientific, Waltham, MA, USA) and then seeded for cell passage or further experiments. Only hMSCs passaged 6–10 were used in our experiments.

Carbonyl cyanide 4-(trifluoromethoxy) phenylhydrazone (FCCP) (Sigma Aldrich, St Louis, MO) was dissolved in DMSO at a stock concentration of 10 mM and stored at −20 °C. FCCP was used at different final concentrations of 0.1, 0.3, 1, 2, 3 μM diluted by hMSC cultured medium. Cells were exposed to different concentrations of FCCP for 20 h; then O_2_ consumption and cellular impedance were measured.

### 2.2. Measurement of O_2_ Consumption

**Figure 1:**
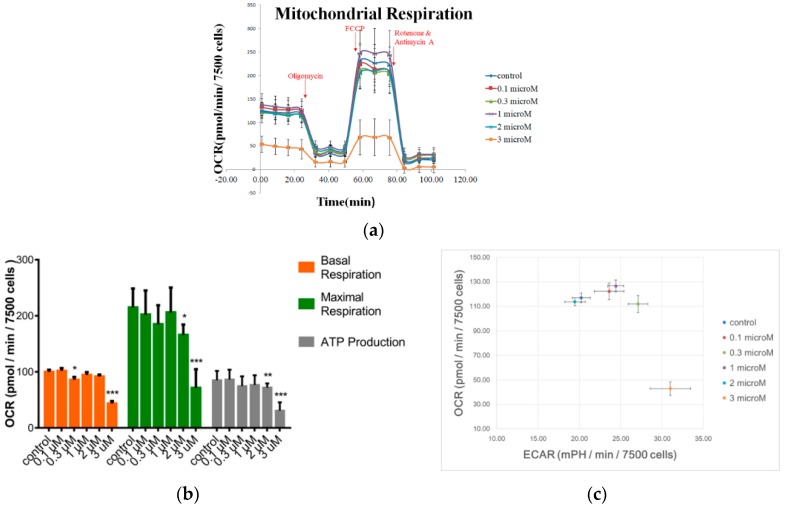
Effect of FCCP on mitochondrial respiration. (**a**) OCR changes in response to ATP synthase inhibitor (1 μM oligomycin), uncoupler (2 μM FCCP), and ETC blockade (1 μM rotenone plus 1 μM antimycin). (**b**) Basal respiration, maximal respiration, and ATP production measured of FCCP-treated hMSCs by Seahorse XF-24 extracellular flux analyzer. The mitochondrial functions were measured after hMSCs were treated with FCCP of 0, (control, *n* = 19), 0.1 (*n* = 11), 0.3 (*n* = 11), 1 (*n* = 24), 2 (*n* = 13), and 3 (*n* = 14) μM for 20 h. Data are presented as the average OCR ± standard error. * *p* < 0.05, ** *p* < 0.01, *** *p* < 0.001. (**c**) The ratio of fundamental respiration. The ratio of OCR to ECAR can indicate overall preference for OXPHOS versus glycolysis. Data are presented as the average OCR or ECAR ± standard error.

For oxygen consumption measurements in hMSCs, cells were seeded at a density of 2.5 × 10^4^ cells/cm^2^ and cultured until approximately 80% confluence before FCCP treatment. After hMSCs were exposed to different concentrations of FCCP for 20 h, the oxygen consumption rate (OCR) and the extracellular acidification rate (ECAR) of cells were measured using a Seahorse XF-24 extracellular flux analyzer (Seahorse Bioscience, Billerica, MA, USA). Both OCR and ECAR were determined as described previously [29]. Basically, the assessment of mitochondrial respiration was performed using a Seahorse XF Cell Mito Stress Test Kit (Seahorse Bioscience, Billerica, MA, USA) which uses inhibitors that specifically target components of electron transport chain (ETC) to quantify key parameters of mitochondrial respiration. As shown in Figure 1a, first, 1 μM oligomycin was injected to inhibit OXPHOS, which was an inhibition of ATP generation and oxygen consumption. Next, 2 μM FCCP was injected to uncouple the ETC, which contributed to an increase of OCR for the maximal respiration measurement. Finally, a 0.5 μM complex of rotenone and antimycin A was injected to inhibit the ETC of Complex I and Complex III, which triggered off the minimum OCR. The mitochondrial functions were analyzed by the specific OCR and expressed as basal respiration, maximal respiration, and ATP production.

### 2.3. Impedance Measurement

The ECIS ZTheta system, electrode arrays, and software were obtained from Applied BioPhysics (Troy, NY, USA). Each 8W1E electrode array consisted of eight wells that were 1 cm in height and 0.8 cm^2^ in area. A small gold sensing electrode (250 μm in diameter) and a much larger gold counter electrode were fabricated on the bottom surface of each well; culture medium was used as the electrolyte. The electrodes were connected through a relay bank to a lock-in amplifier, which measured in- and out-of-phase voltages across the cell-covered electrode. Voltage and phase data were stored and processed with a laptop. These data were then converted to resistance or capacitance treating the cell-electrode system as a series RC circuit. The instrument recorded impedance data at frequencies ranging from 62.5 Hz to 64 kHz. Cells were inoculated into each electrode well at 4 × 10^4^ cells/cm^2^ density and allowed to attach and spread for 24 h before adding FCCP. The confluent cell layer was confirmed by phase-contrast microscopy and also by the impedance measurement of the cell-covered electrode.

### 2.4. Detection of Micromotion

Micromotions of hMSCs are revealed as fluctuations in the measured impedance in the confluent layer. Impedance fluctuation data were acquired after the cells were treated with different concentrations of FCCP for 20 h. To quantify the micromotion of cells via ECIS, data points were sampled from individual electrodes at intervals of 1 sec for a duration of slightly over 0.3 h (1200 data points). Only the first 1024 data points in each sampled electrode were used for data analysis. Each data point consists of the impedance across the electrode both in- and out-of-phase (namely resistance and capacitance reactance) with the applied 4 kHz signal. From data set such as this, one of the algorithms to numerical analyze the cell-generated fluctuations is to calculate the variance for different time periods [24]. Each data point in the 1024-point data set was first divided by the average value of the 1024-point data set. The normalized data set of 1024 points was then split into 256 sets of 4 points each, and the variance was calculated and averaged for all of the sets. The process was then repeated with 8, 16, 32 etc. points in each set, and the average variance is named as Var8, Var16, Var32 etc. In order to represent the resistance fluctuations with a single number, micromotion for each treat group was represented by Var32 and compared with that of the control group.

### 2.5. ECIS Wound Healing Assay

We applied ECIS wound-healing assay to quantify changes of hMSC migration in response to FCCP. In this assay, hMSCs were grown for 24 h until they were confluent in the ECIS wells. Cells were then treated with different concentrations of FCCP for another 20 h. To electrically wound hMSC monolayers, an elevated current pulse of 1.4 mA at 40 kHz was applied for 10 s and caused the death and detachment of cells. The measured impedance dropped to a very low value which is close to the impedance of a cell-free sensing electrode. Following the wounding process, living cells outside the sensing electrode migrated inward to cover the wounding area until the wound was sealed by cells. The ECIS system then switched back to its measurement mode and continuously monitored the wound healing process under different experimental conditions for at least 15 h. To quantify the wound healing migration of hMSCs, the 15-h resistance data measured at 4 kHz after wounding were selected and analyzed. The hill slope was fitted to a sigmoid curve from baseline to plateau. The half-time, T50, was defined as the half-way recovery time from baseline to plateau [23]. It is generally assumed that the rate of wound recovery migration is inversely proportional to T50.

### 2.6. Statistical Analysis

Statistical analysis was performed using Student’s t-test and one-way ANOVA. The level of significance was set at * *p* < 0.05, ** *p* < 0.01, and *** *p* < 0.001. All data are expressed as mean ± standard error.

## 3. Results

### 3.1. Reduction of hMSC Mitochondrial Respiration in Response to FCCP

To identify mitochondrial respiration of hMSCs in response to various concentrations (0, 0.1, 0.3, 1, 2, and 3 μM) of FCCP treatment for 20 h, two metabolic parameters, OCR and ECAR, were measured using Seahorse XF-24 extracellular flux analyzer. Treatment of hMSCs with oligomycin inhibited mitochondrial ATP synthesis and resulted in similar basal OCR (baseline) for all groups except 3 μM, indicating that the lower basal OCR in 3 μM group was attributed to a lower level of coupled mitochondrial respiration (Figure 1a). FCCP treatment following oligomycin resulted in higher OCR increase in 0 to 2 μM groups than in 3 μM group, validating a lower level of maximal mitochondrial activity in 3 μM group. (Figure 1a). Addition of 2 mM FCCP restores proton movement and causes maximal O_2_ consumption. Incubation with rotenone and antimycin completely blocks mitochondrial respiration. The difference between blocked and FCCP-induced OCR is defined as the maximal mitochondrial respiration. A moderately dose-dependent decline of mitochondrial functions induced by FCCP was observed and illustrated in Figure 1b. Compared with the basal OCR of the control cells, the basal OCRs of 0.3, 1, 2, and 3 μM FCCP-treated cells decreased by 14% (*p* < 0.05), 5% (*p* > 0.05), 8% (*p* = 0.08), and 56% (*p* < 0.001) respectively. Since the basal respiration represents the minimal amount of ATP produced by OXPHOS, the inhibition of OXPHOS-based metabolism increases with increasing FCCP concentration. Similarly, the reduction of both maximal respiration and ATP production of the FCCP-treated hMSCs increases with increasing FCCP concentration. More specifically, compared with the maximal respiration of the control cells, the maximal respiration of 0.3, 1, 2, and 3 μM FCCP-treated cells decreased by 14% (*p* = 0.06), 4% (*p* = 0.06), 23% (*p* < 0.05), and 67% (*p* < 0.001) respectively. Furthermore, compared with the OXPHOS-ATP production of the control cells, the ATP production of 0.3, 1, 2, and 3 μM FCCP-treated cells decreased by 13% (*p* = 0.06), 10% (*p* = 0.06), 15% (*p* < 0.01), and 64% (*p* < 0.001) respectively (Figure 1b). Furthermore, since the extracellular acidification rate (ECAR) is a predominant measure of lactic acid during glycolytic energy metabolism, the ratio of OCR and ECAR determines the contribution of OXPHOS and glycolysis energy metabolism [29]. As shown in Figure 1c, only 3 μM-treated cells were inhibited in aerobic metabolism compared with the control group.

### 3.2. Real-Time Monitoring of hMSC Attachment and Spreading

**Figure 2:**
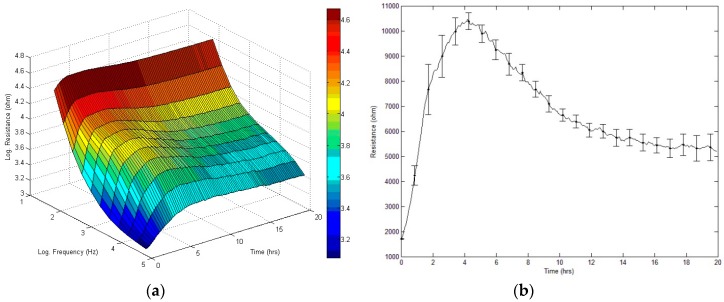
ECIS measurement of hMSC attachment and spreading. At time zero, hMSCs were inoculated in an ECIS well giving a final cell density of 40,000 cells per cm^2^. (**a**) Three-dimensional representation of the changes in resistance as a function of frequency and time during the attachment and spreading of hMSCs on a sensing electrode. (**b**) Changes in resistance as a function of time measured at 4 kHz. Data obtained from eight electrode wells were averaged and presented as mean ± standard error.

Data obtained from a typical 20-h run of hMSC attachment and spreading are presented as a three-dimensional graph to indicate the changes in resistance as a function of frequency and time (Figure 2a). Here the cells were inoculated at time zero and impedance measurements were performed at 11 different frequencies (62.5–64 kHz). Figure 2b shows the resistance data measured at 4 kHz and as a function of time, where 4 kHz is the optimal detection frequency for assessing hMSCs. When cells attach and spread on the sensing electrode, their membranes effectively interfere with the space directly above the electrode available for current flow. This causes an increase in the measured resistance and for hMSCs it generally reaches the highest resistance value 4 h after cell inoculation. Since hMSCs can grow as a confluent monolayer, the measured resistance of the cell-covered electrode was relatively high, approximately 5–6 kΩ at 4 kHz. After the cells are fully spread the measured impedance continues to fluctuate. These fluctuations are the result of the constant motion of the cells altering the current flow in subtle ways. Both the final average impedance values and the fluctuations are characteristic for the cell type and may be used as a cell feature or signature.

### 3.3. Effect of FCCP on the Time Course of Overall Resistance

**Figure 3:**
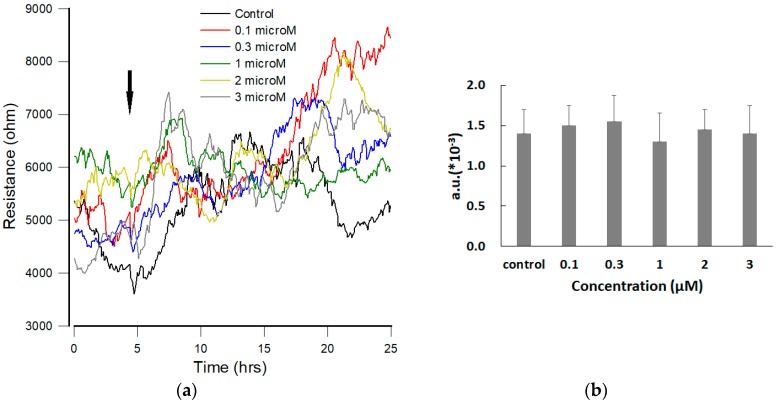
(**a**) Resistance measurements of confluent hMSC monolayers upon addition of different concentrations of FCCP. Cells were inoculated into electrode-containing wells and allowed to develop into confluent layers for approximately 24 h. At the time indicated by the arrow, FCCP diluted in DMSO was added to give the final concentrations of 0.1 μM (red), 0.3 μM (blue), 1 μM (green), 2 μM (yellow), 3 μM (gray), and control (black), and the resultant changes in resistance were followed. Data were collected for 20 h after adding FCCP. Here only the resistance data at 4 kHz were shown. (**b**) Var32 analysis of the normalized resistance data shown in Figure 4a. Dose dependent effect of FCCP on the overall resistance of hMSCs is not significantly observed. Values shown are mean ± standard error (*n* = 8 for each concentration of FCCP).

The effect of various concentrations (0, 0.1, 0.3, 1, 2, and 3 μM) of FCCP on the overall resistance of the hMSC monolayer was monitored for 20 h. Impedance measurements were also performed at 11 different frequencies (62.5 Hz–64 kHz). Sixteen electrodes were followed one after another with 11 data points of each well requiring 10 s. Although the impedance of each electrode well was set to be measured every 160 s, fluctuations were observed on each curve at different level. The typical data for each FCCP concentration were presented as the measured resistance at 4 kHz and as a function of time (Figure 3a). Surprisingly, in terms of the average value of the overall resistance, there were no evident differences among the six time-series curves (data not shown), indicating that hMSCs maintain stable morphology during the 20-h FCCP exposure. In addition, no significant differences of the Var32 values of these time series data were observed (Figure 3b).

### 3.4. Effect of FCCP on hMSC Micromotion

**Figure 4:**
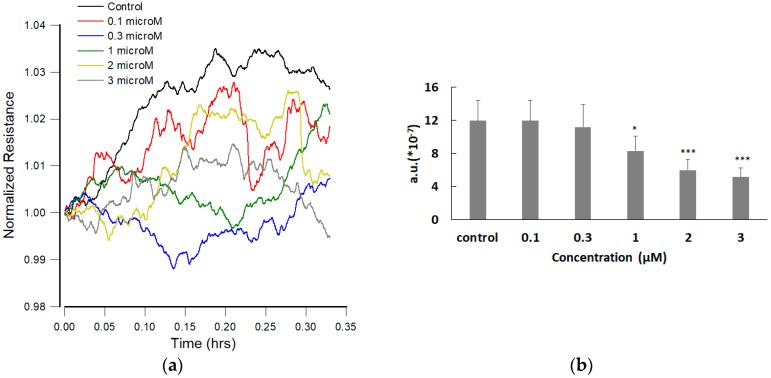
(**a**) Normalized resistance data recorded 20 h after the addition of medium containing FCCP or medium alone to a confluent hMSC layer to give the final concentrations of 0.1 μM (red), 0.3 μM (blue), 1 μM (green), 2 μM (yellow), 3 μM (gray), and control (black). Each curve consists of 1200 data points taken at 1-s intervals. Here only the first 1024 data points in each curve were used to calculate Var32 values. (**b**) Var32 analysis of the normalized resistance data shown in Figure 4a. Dose dependent effect of FCCP on hMSC micromotion is observed. Values shown are mean ± standard error (*n* = 10 for each concentration of FCCP). * *p* < 0.05, *** *p* < 0.001.

Previous studies have suggested that cell movements in confluent layers detected via ECIS can be correlated with cell metabolic activity [20,24]. In addition, fluctuation analysis of the measured resistance time series could be an effective assessment of cytotoxicity if data acquisition had been faster [25,26]. To characterize the micromotion profiles of hMSCs in response to different concentrations of FCCP, the rapid time series (RTC) measurement at 4 kHz was performed after 20-h FCCP exposure of hMSCs. For detection of cell micromotion, impedance data of each well were taken every second until 1024 points had been acquired and then another well was measured. The time-series data were normalized and numerically analyzed by calculating Var32 as we previously described [24,25]. Briefly speaking, the data set of 1024 points was first split in 32 equal sets with 32 points each, and the variance of the 32 points in each set was calculated and averaged for all the sets to obtain the Var32. Figure 4a shows a graph of typical resistance data normalized to its value at the start of each run. While there are no significant differences of the average resistance value among the six time-series curves (data not shown), Var32 values of these time series data show a dose-dependent drop and are capable of detecting the effect of 1 μM FCCP on the hMSC monolayer (Figure 4b).

### 3.5. Effect of FCCP on hMSC Wound Healing Migration

**Figure 5:**
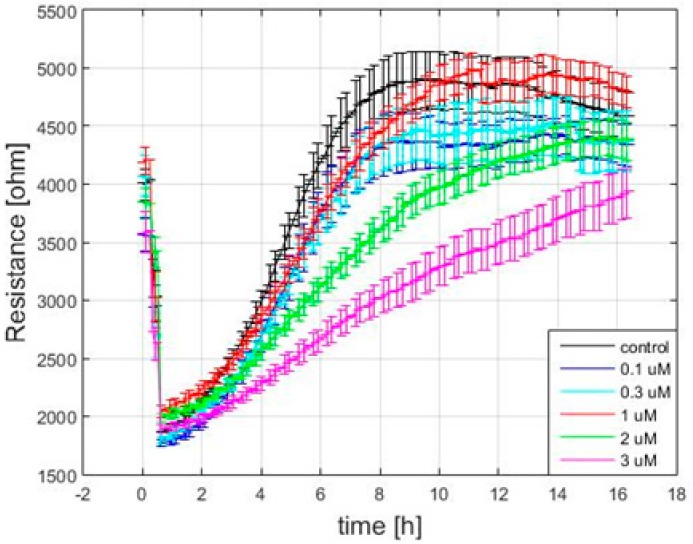
The resistance time series of wound healing migration measured at 4 kHz. The measurements were taken 20 h after hMSCs were exposed to 0.1 (*n* = 20), 0.3 (*n* = 32), 1 (*n* = 28), 2 (*n* = 32), 3 (*n* = 25) μM of FCCP and culture medium as control (*n* = 36).

**Figure 6:**
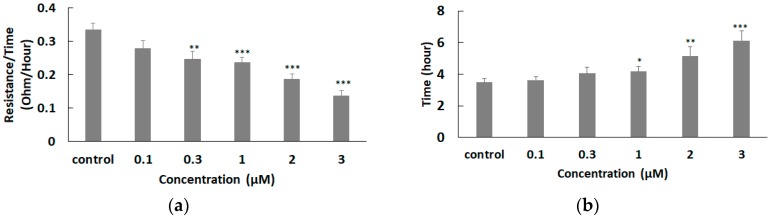
(**a**) Hill slope and (**b**) T50 values obtained from the ECIS wound healing assay of hMSCs in response to various concentrations of FCCP. Significance was calculated by comparing each experimental group with the control group. Values shown are mean ± standard error. * *p* < 0.05; ** *p* < 0.01; *** *p* < 0.005.

**Figure 7:**
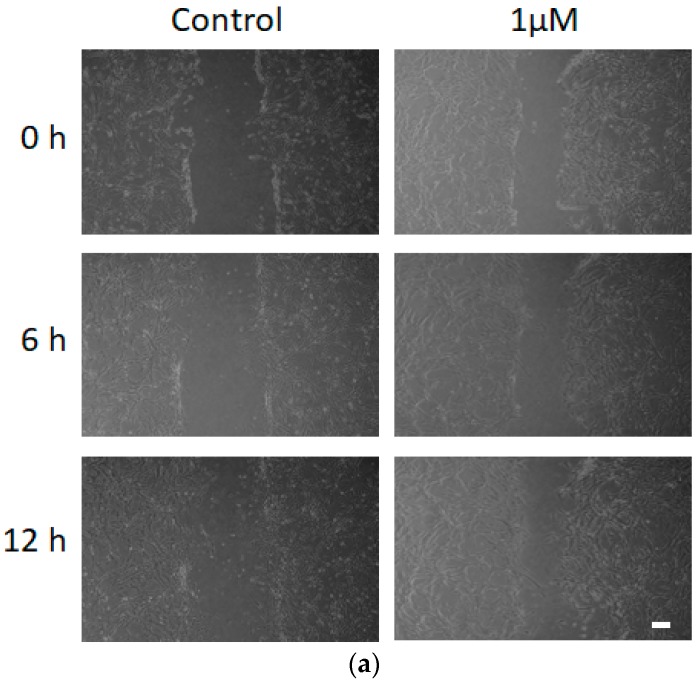

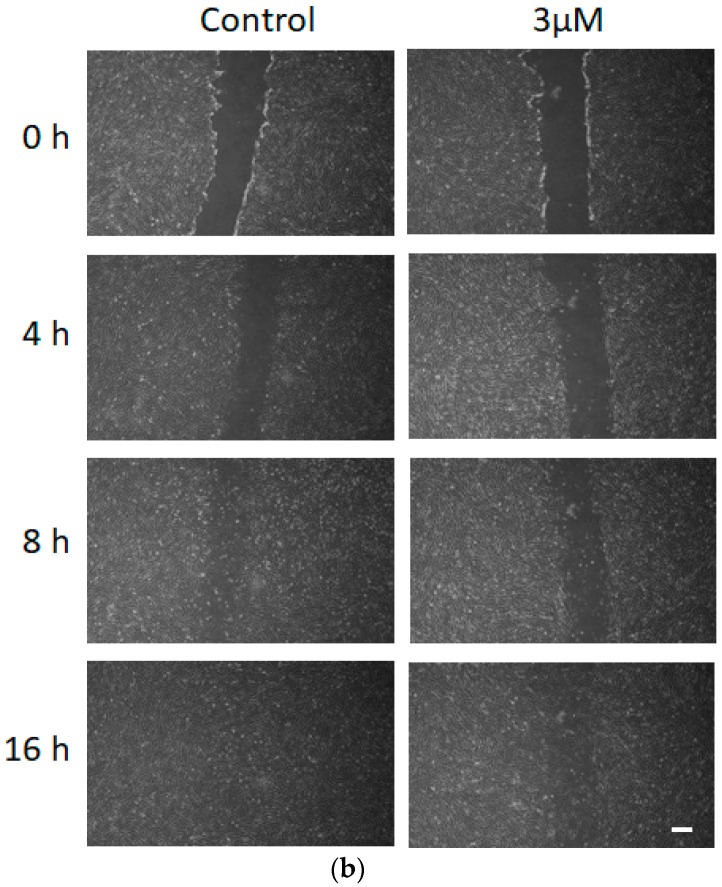
Time-lapsed images of scratch wound-induced migration of hMSCs after they were cultured in the culture medium without (control) and with (**a**) 1 μM and (**b**) 3 μM FCCP for 20 h. Gaps were created by gently dragging a 200 µL pipette tip across the bottom of the 35-mm cultured dishes. Scale bar is 100 μM in length.

To further understand the effect of FCCP on the migratory ability of a hMSC monolayer, ECIS wound-healing migration measurements were taken from cell-covered electrodes 20 h after exposure to different concentrations of FCCP. The measured resistance data at 4 kHz were selected at 15 h post the wounding process, and the recovery curve was fitted to a sigmoid curve from baseline to plateau. As shown in Figure 5, cells were electrically wounded at the time point of 0.5 h, and data acquisition was briefly suspended for 6 min. After wounding, resistance values after wounding decreased to approximately 1.7–2 kΩ, which is close to the value of a cell-free electrode, indicating cell death and detachment on the sensing electrodes. Data acquisition was then restarted at time point of 0.6 h. It is generally assumed that the time required for cells to migrate inward until the wound is closed by cells depends on the size of the sensing electrode area and their migration rate [22]. T50 denotes the half-way recovery time of the measured resistance time course from baseline to plateau. Therefore, migration rate of cells can be determined by dividing the electrode radius (125 μm) with two times of T50 [23]. In Figure 5, statistically, there were no significant differences of the average resistance values among the six time-series curves before wounding. However, recovery curves of 1, 2, and 3 µM FCCP treatment after wounding demonstrated different profiles compared to control, indicating the inhibitory effects of 1–3 µM FCCP on hMSC wound-healing migration. To quantify the wound healing data presented in Figure 5, hill slope was fitted and T50 was calculated for each FCCP concentration (Figure 6). After hMSCs were exposed to different concentrations of FCCP for 20 h, a dose-dependent decrease in hill slope and a dose-dependent increase in T50 were observed (Figure 6). Taken together, consistent with the findings of mitochondrial functions and cell micromotion, these results suggest that the wound-healing migration rate of hMSCs decreases with increasing FCCP concentration. Moreover, both measures of hill slope and T50 are respectively capable of detecting the effect of 0.3–3 μM and 1–3 μM FCCP on the hMSC migration. On the contrary, observations from scratch wound-induced migration assay showed that, compared with hMSCs in the culture medium (control, 14 ± 2 μm/h, *n* = 5), migratory difference was not significantly distinguished for cells exposed to 1 μM FCCP for 20 h (Figure 7a, 13 ± 2 μm/h, *n* = 3) but for cells exposed to 3 μM FCCP for 20 h (Figure 7b, 8 ± 1 μm/h, *n* = 3). This result confirms the high sensitivity of the ECIS micromotion and wound-healing migration assays.

## 4. Discussion

Mitochondria plays an important role in cell metabolism and one of its key functions is the generation of ATP. FCCP, which is a classical uncoupler of OXPHOS, has been extensively used to induce mitochondrial dysfunction through the disruption of the mitochondrial membrane potential. Using Seahorse XF-24 extracellular flux analyzer, compared with the control group, both maximal respiration and ATP production of 2 μM and 3 μM FCCP-treated hMSCs were shown to significantly decrease (Figure 1). It is worth noting that no significant changes of 20-h hMSC proliferation were detected after cells were treated with 0, 0.1, 0.3, 0.5, 1, 2, and 3 μM FCCP (data not shown). Taken together, these results indicate that glycolysis is essential for 2 or 3 μM FCCP-treated hMSC bioenergetics due to its low mitochondrial respiratory capacity. Sensitively detecting the cytotoxic effects of low levels of FCCP on stem cell metabolism is difficult since FCCP has been known as a mild uncoupler [30]. Recent in vitro studies show that cultured cells resist treatment with CCCP and FCCP and survive, even after the loss of mitochondrial membrane potential and termination of mitochondrial ATP production [31,32]. These observations indicate that, depending on the exposure time and the concentration of the compounds, cells are able to adapt by developing protective mechanisms against CCCP/FCCP-induced apoptosis. Uncouplers like CCCP and FCCP seem to reverse the physiological role of oxygen and create a hypoxic environment, leading to the loss of cytoskeletal features and formation of blebs [33]. It has been reported that for rhabdomyosarcoma (RD) cells, there was an increase in the depolarized cell population with increasing FCCP concentrations. In addition, RD cells treated with 20 μM FCCP for 10, and 24 h were elongated rather than polyhedral and were generally smaller (attenuated) [33]. Taken together, these results indicate FCCP-mediated uncoupling of OXPHOS can reduce ATP production, and consequently attenuate hMSC migration. It is likely that in vitro stem cell behaviors such as cell micromotion and wound healing migration may be valuable predictors of stem cell metabolism in response to CCCP and FCCP.

In ECIS measurement, the resistances of the sensing electrodes increase dramatically when cells attach and spread upon electrode surfaces (Figure 2). The motion of the cells manifests itself as a fluctuation in the impedance measurement even after they form a confluent monolayer. The magnitude of the motions detected by this method is of the order of nanometers. We refer to this motion as micromotion, and in the past years, we have shown that this is the end result of a chain of complex reactions in the cells. By studying micromotion under different external conditions, we have obtained evidence that it is correlated with the cell’s metabolic activity [24,25,26]. While there are no significant differences of the overall resistance among the six time-series curves (0, 0.1, 0.3, 1, 2, and 3 μM), during or after the 20-h exposure of FCCP (Figure 3 and Figure 4a), by calculating Var32 values of these time series we are able to detect the effects of FCCP on the hMSC monolayer at concentrations down to 1 μM (Figure 4b). It is probable that the effects of FCCP may be discernable at FCCP levels lower than 1 μM using other analytical methods including the power spectrum, the Hurst and detrended-fluctuation-analysis exponents, and the first zero and first 1/e crossings of the autocorrelation function [26]. While most published works with impedance-based cellular assays use only average impedance values, we demonstrate that fluctuation analysis of the impedance time series measured by ECIS provides a more sensitive probe. Our future research in this area will focus on stem cell behaviors under different environmental (e.g., toxin, oxygen, and glucose) conditions.

We have previously reported the use of ECIS wound healing assay to investigate cytotoxic effects of andrographolide on U-87 MG glioblastoma cell migration [28]. In the present study, we have used ECIS to assess hMSC wound-healing migration in response to low levels of FCCP and demonstrated the effect of FCCP-induced mitochondrial dysfunction on cell migration rate in a dose-dependent manner. While Seahorse XF-24 extracellular flux analyzer can detect the effect of FCCP on maximal respiration and ATP production of hMSCs at 2 μM and 3 μM concentrations (Figure 1), the Var32 calculation of the time-series resistances measured at 4 kHz can detect the effect of FCCP at concentrations as low as 1 μM (Figure 4). For wound healing migration, the recovery resistance curves were fitted by sigmoid curve and the hill slope showed a significant concentration-dependent decrease from 0.3 μM to 3 μM, indicating a decrease in cell migration rate (Figure 5 and Figure 6). Moreover, a dose dependent increase of the T50, the half time of wound recovery migration, was significantly observed from 1 μM to 3 μM. Together, these results suggest that partial uncoupling of mitochondrial oxidative phosphorylation reduces micromotion and wound healing migration of hMSCs. Short term (20-h) exposure of 2 or 3 μM FCCP causes significant impact on migratory behaviors of hMSCs.

## 5. Conclusions

In this study, we applied ECIS to investigate the effect of FCCP-induced uncoupling of mitochondrial OXPHOS on hMSC migration. We demonstrate that mitochondrial dysfunction of hMSCs is associated with decreased ATP production from OXPHOS and decreased migratory capacity including cell micromotion and wound healing migration. The ECIS method used in this study offers a simple and sensitive approach to investigate stem cell migration and its regulation by mitochondrial dynamics. Changes of Var32, hill slope and T50 may provide sensitive indicators of uncoupling in response to chemical exposure.
